# In vitro study on aspects of molecular mechanisms underlying invasive aspergillosis caused by gliotoxin and fumagillin, alone and in combination

**DOI:** 10.1038/s41598-020-71367-2

**Published:** 2020-09-02

**Authors:** Loganathan Gayathri, Mohammad A. Akbarsha, Kandasamy Ruckmani

**Affiliations:** 1 Department of Pharmaceutical Technology, University College of Engineering, Anna University-BIT Campus, Tiruchchirappalli, Tamil Nadu 620024 India; 2 Centre for Excellence in Nanobio Translational Research (Autonomous), University College of Engineering, Anna University-BIT Campus, Tiruchchirappalli, Tamil Nadu 620024 India; 3 Department of Biotechnology and Bioinformatics, Holy Cross College (Autonomous), Tiruchchirappalli, Tamil Nadu 620002 India; 4 National College (Autonomous), Tiruchchirappalli, Tamil Nadu 620001 India; 5 Mahatma Gandhi-Doerenkamp Centre for Alternatives, Bharathidasan University, Tiruchchirappalli, Tamil Nadu 620 024 India

**Keywords:** Microbiology, Risk factors

## Abstract

Gliotoxin (GT) and fumagillin (FUM) are mycotoxins most abundantly produced by *Aspergillus fumigatus* during the early stages of infection to cause invasive aspergillosis (IA)*.* Therefore, we hypothesized that GT and FUM could be the possible source of virulence factors, which we put to test adopting in vitro monoculture and the novel integrated multiple organ co-culture (IdMOC) of A549 and L132 cell. We found that (i) GT is more cytotoxic to lung epithelial cells than FUM, and (ii) GT and FUM act synergistically to inflict pathology to the lung epithelial cell. Reactive oxygen species (ROS) is the master regulator of the cytotoxicity of GT, FUM and GT + FUM. ROS may be produced as a sequel to mitochondrial damage and, thus, mitochondria are both the source of ROS and the target to ROS. GT-, FUM- and GT + FUM-induced DNA damage is mediated either by ROS-dependent mechanism or directly by the fungal toxins. In addition, GT, FUM and GT + FUM may induce protein accumulation. Further, it is speculated that GT and FUM inflict epithelial damage by neutrophil-mediated inflammation. With respect to multiple organ cytotoxicity, GT was found to be cytotoxic at IC_50_ concentration in the following order: renal epithelial cells < type II epithelial cells < hepatocytes < normal lung epithelial cells. Taken together, GT and FUM alone and in combination contribute to exacerbate the damage of lung epithelial cells and, thus, are involved in the progression of IA.

## Introduction

*Aspergillus fumigatus,* the abundantly distributed saprophytic fungus, is a weak pathogen. Generally, *A. fumigatus* releases air-borne, dormant, buoyant microscopic conidiospores in copious amounts, so that a person would potentially inhale hundreds of spores every day^1^. After successful permeation of airway mucosa, the spores encounter the airway epithelial cells where the first line immune response of the host is likely to begin^1,2^. After vigorous hyphal growth for 24 h, the fungus may produce some secondary metabolites that breach the epithelial/endothelial barrier. Once the epithelial layer of the alveoli is damaged, the fungus enters the endothelium of blood vessels to become an angiotrophic fungus whereupon the disease becomes invasive and spreads to other organs^2,3^. However, immunocompetent healthy individuals are able to deal with the fungal conidia by the several immune mechanisms that prevent the germination and growth of hyphae whereas in immunocompromised persons conidial germination and mycelial development in the lung epithelial cells can cause severe/fatal disease called Invasive Aspergillosis (IA)^1,3^.

Reports have shown that IA is now a major cause of death at leukemia treatment, bone marrow transplantation and solid-organ transplantation centers and it is responsible for 30% of fungal infections in patients dying of cancer wherein the mortality rate of leukemia patients is 80 to 90%, even when given antifungal therapy^2,3^. Thus, anti-*A*. *fumigatus* therapy remains challenging as a consequence of high mortality from IA^4^. One of the major reasons for this failure could be poor understanding of the colonization, pathobiology and virulence factors of *A*. *fumigatus* because every pathogen is capable of developing strategies to disseminate hyphal growth and evade host immune surveillance during infection^3,4^. In order to fight against the first-line host innate immune response, *A*. *fumigatus* adopts a strategy that involves discharge of mycotoxins to damage the epithelial/endothelial barriers of the respiratory tract. For instance, it has been reported that *A*. *fumigatus* relies on secreted proteases, lipases, and/or toxins for their survival benefits but none of these have been explored for pathogenesis of IA except mycotoxins^5^.

Mycotoxins are secondary metabolites of fungi which are not vital for their lifecycle but offer competitive survival advantage over the host immunity. *A. fumigatus* produces a variety of mycotoxins/secondary metabolites such as gliotoxin, fumagillin, fumitremorgin, verruculogen, restrictocin, helvolic acid, etc. Although the pathogenicity of IA is multifactorial, gliotoxin has been proved to be a virulent factor of *A*. *fumigatus* but the specific roles of the other mycotoxins are not well defined^1,3,4^. Indeed, the combined action of two or more of these mycotoxins may produce synergistic effects against the host’s defense mechanisms. In the light of the fact that two or more mycotoxins would interact synergistically or additively and produce more serious adverse effects than single compounds^6,7^, it is pertinent that this issue in respect of highly virulent mycotoxins produced by A. *fumigatus* is worthy of being addressed.

Gliotoxin (GT), a hydrophobic metabolite, belongs to the class of epipolythiodioxopiperazine compounds characterized by a quinoid moiety and disulfide bridge across the piperazine ring which is essential for their toxicity^8^. GT is a well-studied immune-suppressive mycotoxin that is produced against the first line immune response of epithelial barrier of the host. GT is known to induce apoptosis in leukocytes, and inhibit phagocytosis, respiratory burst, and T-cell and B-cell responses stimulated by the host. Also, GT has received considerable attention as a pathogenic and putative virulence factor as revealed in the following observations: (i) GT was detected in the lung and serum of cancer patients suffering from IA as well as in mice with experimentally induced IA (Table 1); (ii) up to 93% of *A. fumigatus* strains isolated from cancer patients suffering from IA produce GT^9^; and (iii) GT was found to be produced much faster at 37 °C under high levels of oxygen which is close to that of the environment of host’s lung^10^.

**Table 1:** Summary of Serum levels of GT in mice and human.

Mycotoxin	Level of GT in lungs	Level of GT in serum	Experimental model	Reference
GT	3,976 ± 1,662 ng/g	36.5 ± 30.28 ng/ml	Mice	^2^
GT	–	166 to 785 ng/ml	Patients with cancer	^2^
GT	40–63 µg/kg (sputum)	33–47 µg/kg	Patients with pulmonary aspergillosis	^11^
Bis(methylthio)gliotoxin	0.19–13.68 mg/L (bronchoalveolar lavage or sputum)	–	Patients with IA	^12^
GT	–	0.19–0.25 µg/ml	Patients with high risk for IA	^13^
Bis(methylthio)gliotoxin	–	0.46–49.84 µg/ml	Patients with high risk for IA	^11^

Fumagillin (FUM) is a cyclohexane derivative, and identified as a potent angiogenesis inhibitor that reduces the proliferation of endothelial cells for blood vessel formation to avoid infiltration of host’s immune cells to the site of infection^14^. Further, FUM is also involved in IA during hyphal invasion and thereby damage the epithelial layer, inhibit neutrophils and slow down beating of cilia of ciliated epithelial cells^14–17^. In fact, *A. fumigatus* produces both GT and FUM during the early stages of IA, within 30 to 72 h^14,15^. This may facilitate the fungus escape the immune surveillance and propagate the disease so as to become invasive. Despite the fact that the fungal virulence/pathogenicity is mediated by mycotoxins, the relatively unexplored frontier of the lung epithelial interface of either of the mycotoxins alone and/or the two in combination is an untouched medically important area of research that may open up avenues to find new therapies for IA^18^.

Therefore, in this study we aimed at finding the molecular mechanism by which GT and FUM alone, as well as in combination, affect the epithelial cell barrier of the lung, adopting in vitro approach. Human origin A549 (lung carcinoma**)** and L132 (normal) lung epithelial cells that are the most established models to study IA were chosen to explore cytotoxicity, genotoxicity, cell cycle arrest, mechanism of cell death and pro-inflammatory responses against GT and FUM individually as well as in combination. As the disease progresses to invasiveness, we further analyzed organ-specific toxicity of GT in A549 (lung), L132 (lung), HepG2 (liver) and HEK2913 (kidney) epithelial cells by adopting a novel co-culture technique, integrated discrete multiple organ co-culture (IdMOC).

## Results

### Cytotoxic effect of GT and FUM alone and in combination on lung epithelial cell

**Figure 1:**
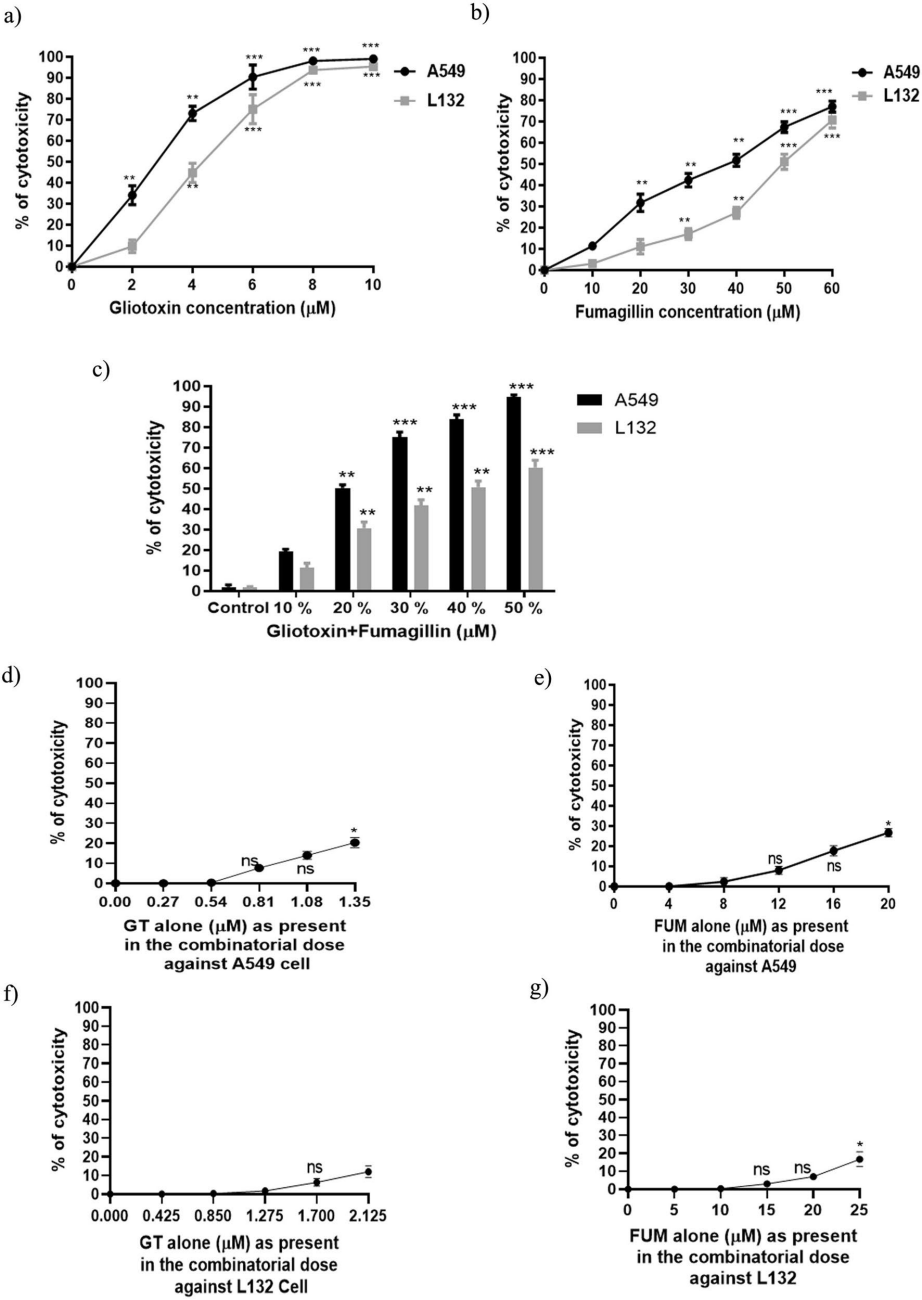
Cytotoxic effect of GT and FUM alone and in combination against A549 and L132 cells following 24 h of exposure. (**a**) Dose–response curve of GT at different concentrations. (**b**) Dose–response curve of FUM at different concentrations. (**c**) Combined cytotoxicity of GT + FUM. (**d**) Dose–response curve of GT alone as present in the combinatorial dose for A549 cell. (**e**) Dose–response curve of FUM alone as present in the combinatorial dose for A549 cell. (**f**) Dose–response curve of GT alone as present in the combinatorial dose for L132 cell, (**g**) Dose–response curve of FUM alone as present in the combinatorial dose for- L132 cell. Data are expressed as mean ± SD of three independent experiments for each dose point (n = 18 for panels (a, d, e, f, g), n = 21 for panel b, n = 36 for panel c). Significance between control and treatment groups was tested by adopting two way ANOVA (ns = not significant, *p < 0.5, **p < 0.01 and ***p < 0.001).

Exposure to GT caused dose-dependent increase in cytotoxicity at the concentration range 0–10 μM and the measured IC_50_ was 2.7 and 4.25 μM for A549 and L132, respectively (Fig. 1a). As shown in Fig. 1b, FUM at the concentration range 0–60 μM resulted in significant increase of cytotoxicity in dose-dependent manner, and the IC_50_ was 40 μM and 50 μM for A549 and L132, respectively. Based on these individual IC_50_ values cells, GT and FUM were combined at 10%, 20%, 30%, 40% and 50% of the respective IC_50_ concentrations as shown in Fig. 1c. The combination of GT and FUM at 20% of the respective IC_50_ (GT 0.54 µM + FUM 8 µM) against A549 cells and 40% of the respective IC_50_ (GT 1.7 µM + FUM 20 µM) against L132 cells significantly increased cytotoxicity to 50%. Figure 1d–g show that individual doses of GT and FUM alone, at the precise concentrations used in combinatorial experiment could not significantly increase 50% cytotoxicity against A549 and L132 cells. This is evidence to the effect that the combinatorial effect is only because of the combination of GT + FUM and not due to individual doses in the combination of GT and FUM against A549 and L132 cells. Also, according to Table 2, the combinations of GT + FUM used in the study showed synergistic interaction at the particular ratio. Therefore, we have used the following dose for treatment purpose throughout the study: IC_50_ concentration of GT (2.7 µM), FUM (40 µM) and GT + FUM (0.54 µM + 8 µM) against A549 and IC_50_ concentration of GT (4.25 µM), FUM (50 µM) and GT + FUM (1.7 µM + 20 µM) were used against L132 cells.

**Table 2:** Synergistic interaction of GT and FUM in A549 and L132 cells.

Mycotoxin	Cell line	Dose effect parameter			CI values of combinatorial dose				
		D_m_ (µM)	m	r	10%	20%	30%	40%	50%
GT	A549	2.51	3.18	0.98					
FUM	A549	35.56	1.54	0.99					
GT	L132	3.86	3.26	0.99					
FUM	L132	49.88	2.75	0.99					
GT + FUM	A549	7.96	2.63	0.97	0.44	0.44	0.41	0.40	0.269
GT + FUM	L132	20.83	1.56	0.99	0.43	0.55	0.72	0.84	0.91

Dm and m values are used for calculating the CI value, i.e., CI < 1, CI = 1 and CI > 1 represent synergism, additivity and antagonism, respectively. Here, Dm, m and r stand for median-effect dose, kinetic order and regression coefficient of the fitting function. The ratios of GT + FUM is 1:14.8 and 1:11.76 against A549 and L132, respectively. CompuSyn software was used for calculation and simulation.

### Effect of GT and FUM alone and in combination on induction of reactive oxygen species (ROS) and oxidative stress gene expression in lung epithelial cells

**Figure 2:**
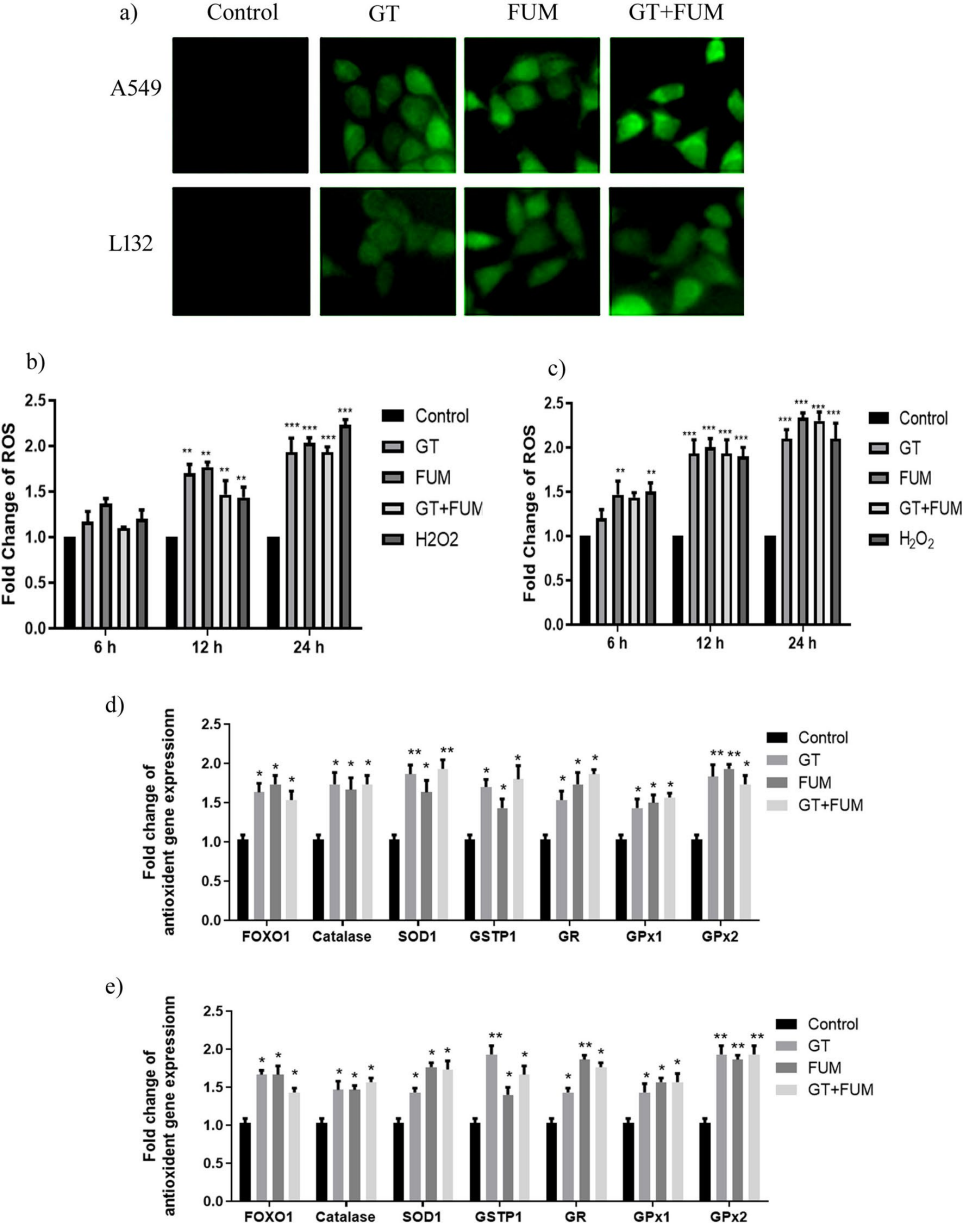
Effect of GT& FUM alone and in combination on induction of reactive oxygen species (ROS) and oxidative stress gene expressions. (**a)** Representative images of ROS generation after 24 h of exposure to GT, FUM and GT + FUM in A549 and L132 cells. (**b**, **c**) Quantification of intracellular ROS generation after 6 h, 12 h and 24 h of exposure to GT, FUM and GT + FUM in A549 and L132 cells, respectively. ROS level is expressed as fold change in the fluorescence in comparison with control (untreated). (**d**, **e**) Expression levels of oxidative stress related genes in A549 and L132 cells, respectively. Data are expressed as mean ± SD of three independent experiments for each dose point (n = 45 for panels b & c, n = 84 for panels **d** and** e**). Significance between control and treatment groups was tested by adopting two way ANOVA (*p < 0.5 **p < 0.01 and ***p < 0.001).

The ability of GT and FUM, alone and in combination, to induce oxidative stress was evaluated. Accumulation of ROS resulted in oxidation of H_2_-DCF to become highly fluorescent DCF, which was observed as green fluorescence in both A549 and L132 cells (Fig. 2a). The results in Fig. 2b and c demonstrate time-dependent increase of ROS in both A549 and L132 cells. H_2_O_2_ was used as the positive control for ROS, and the significant increase of ROS was compared with data of test mycotoxins.

Further, we analyzed the changes in transcript levels of antioxidant/stress-responsive genes such as Forkhead Box O1 (FOXO1), Catalase, Superoxide dismutase 1 (SOD1), Glutathione S-Transferase P1 (GSTP1), Glutathione reductase (GR), Glutathione peroxidase 1 (GPx1) and Glutathione peroxidase 2 (GPx2). As shown in Fig. 2d and e, expression of stress-responsive transcription factor FOXO was elevated to significant level and, consequently, the expression of anti-oxidant genes that are related to FOXO transcription are also significant in A549 and L132 cells exposed to GT, FUM and GT + FUM for 24 h.

### Effect of GT and FUM alone and in combination, on mitochondrial trans-membrane potential (ΔΨm)

**Figure 3:**
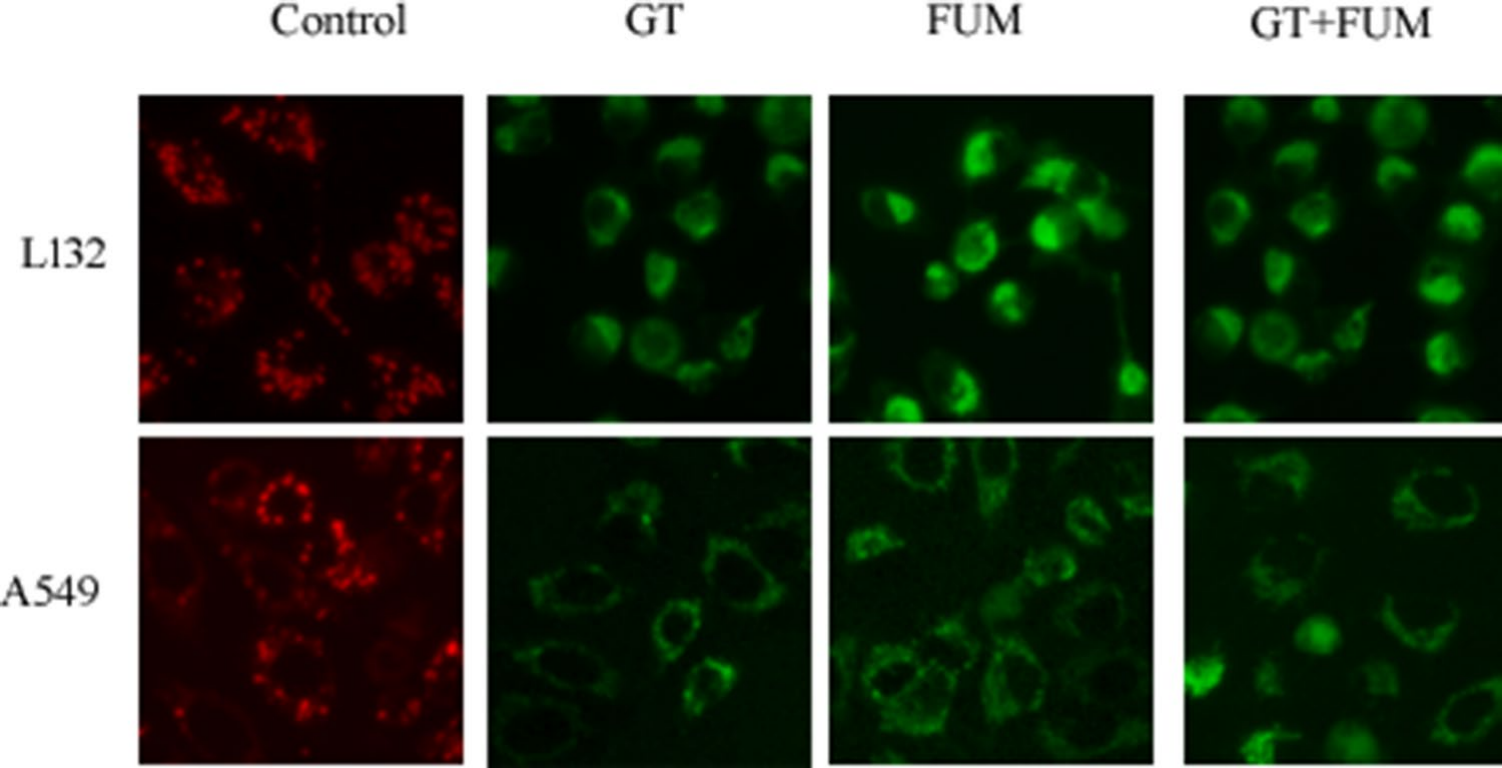
Photomicrographs of L132 and A549 cells showing the effect of GT, FUM alone and in combination on the change in mitochondrial trans-membrane potential at 24 h. Red color indicates high mitochondrial membrane polarization in control (untreated); Green color indicates depolarization of mitochondria in mycotoxin treated cells. Results are representative of three independent experiments.

JC-1 is a probe which indicates loss of ΔΨm by shift in fluorescence emission from red to green. As shown in Fig. 3, control (untreated) cells fluoresced red and cells treated with GT, FUM and GT + FUM fluoresced only green indicating that ΔΨm was lost completely in both A549 and L132 cells within 24 h.

### Evidence for accumulation of unfolded protein on treatment of GT, FUM and in combination in lung epithelial cells

**Figure 4:**
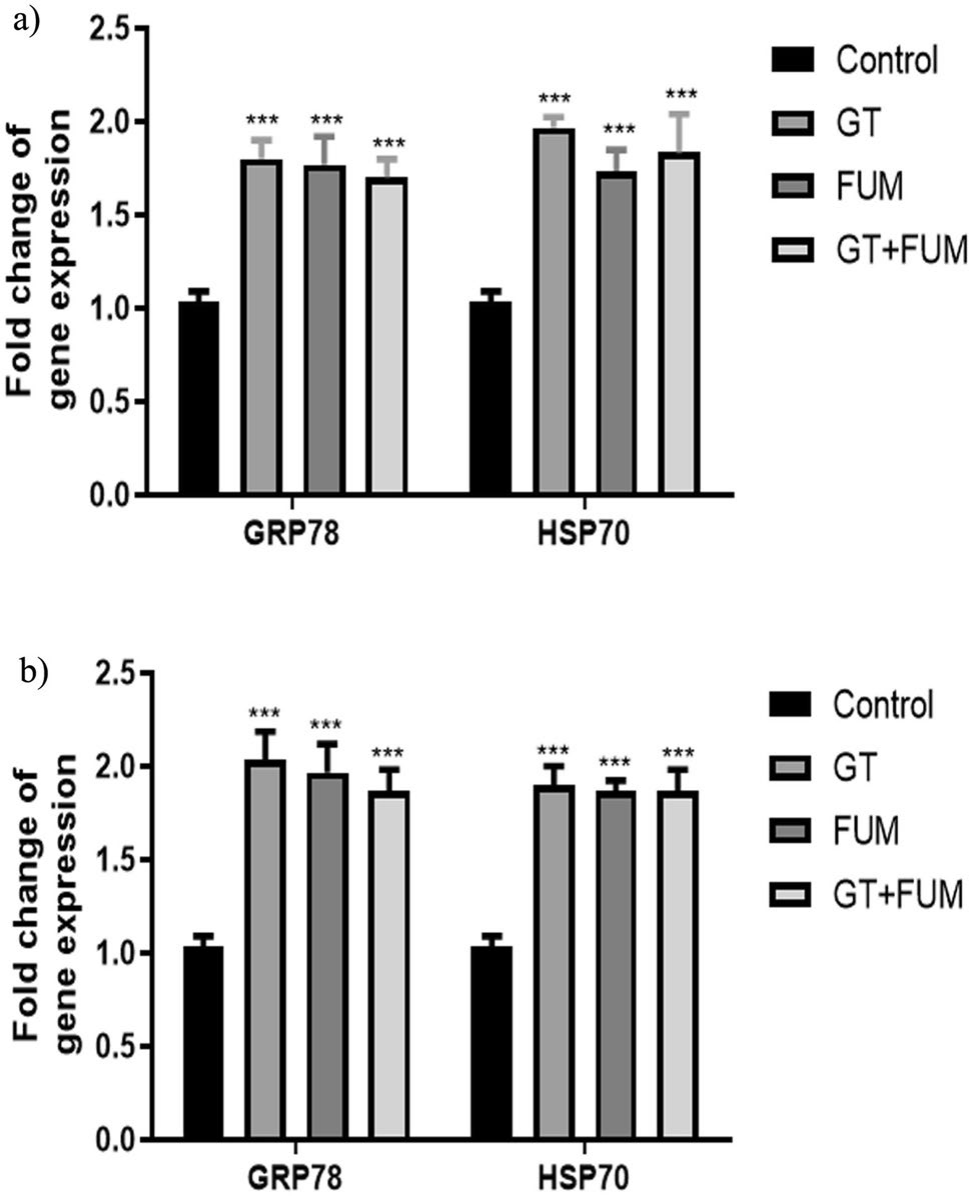
Effect of GT & FUM alone and in combination on gene expression of GRP78 and HSP70 chaperons. (**a**, **b**) Expression levels of GRP78 and HSP70 in A549 and L132 cells, respectively, after 24 h. Data are expressed as mean ± SD of three independent experiments for each dose point (n = 24 for panels a & b). Significance between control and treatment groups was tested by adopting two way ANOVA (***p < 0.001).

Induction of GRP78 and HSP70 transcript levels was evaluated to confirm the accumulation of unfolded proteins in the lumen of the endoplasmic reticulum (ER). Figure 4a and b revealed significant increase in expression of GRP78 and HSP70 chaperons in A549 and L132 cells exposed to the respective concentrations of GT, and FUM each alone and in combination for 24 h. This confirms that GT and FUM each alone and in combination may increase the level of unfolded protein inside the cells and thus pose a chance to induce ER stress.

### Genotoxicity of GT and FUM alone and in combination revealed in comet assay

**Figure 5:**
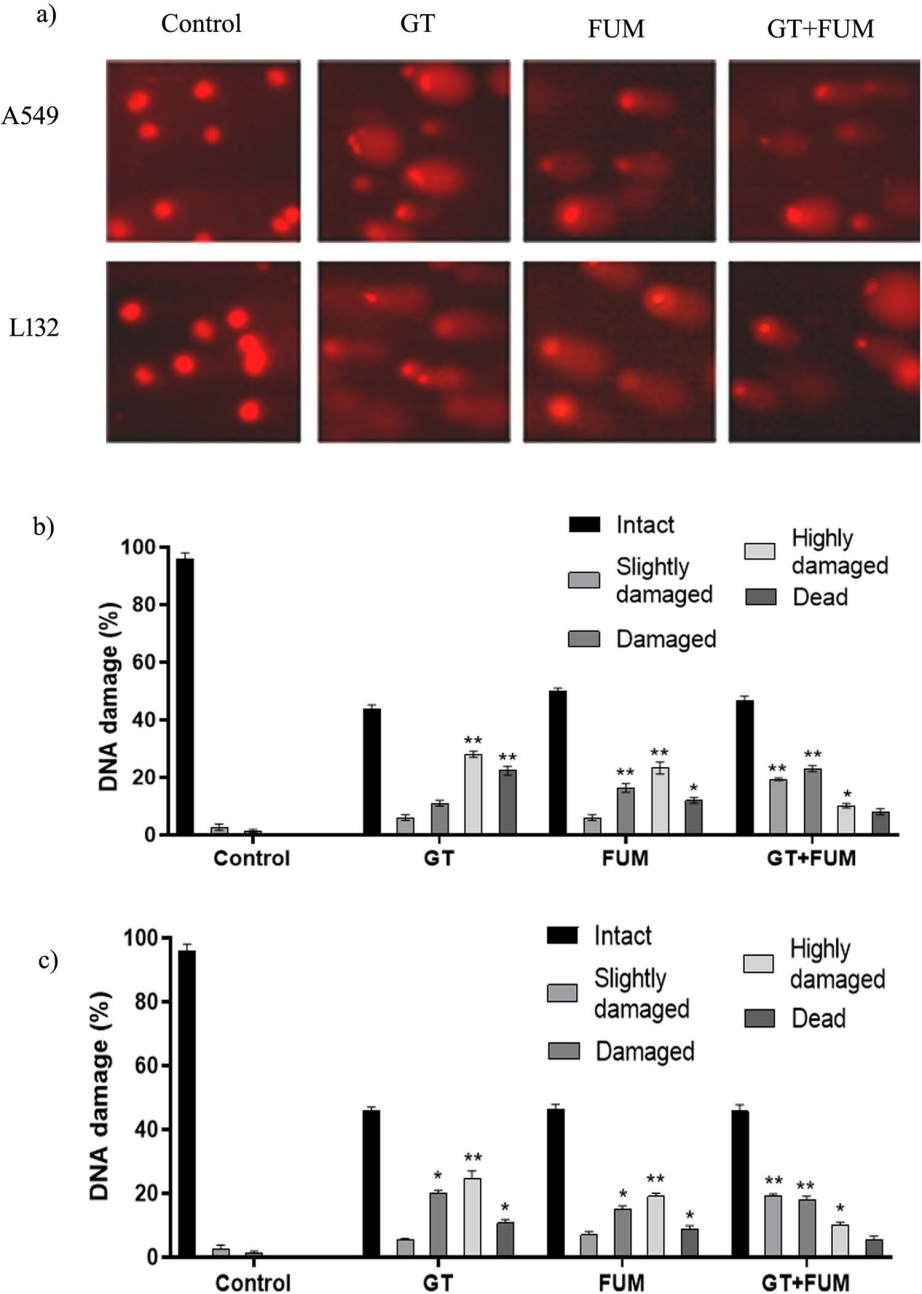
Assessment of DNA damage induced by GT & FUM alone and in combination after 24 h treatment in A549 and L132 cells. (**a**) Representative images of A549 and L132 cells showing DNA damage. (**b**, **c**) DNA damage (intact—0 to 20%, slightly damaged—20 to 40%, damaged—40 to 60%, highly damaged—60 to 80%, and dead—80 to 100%) as defined according to the DNA in the tail of A549 and L132 cells, respectively. Data are expressed as mean ± SD of three independent experiments for each dose point (n = 60 for panels **b** and **c**). Significance between control and treatment groups was tested by adopting two way ANOVA ((*p < 0.5, **p < 0.01).

As shown in Fig. 5a, both GT and FUM caused significant damage to DNA wherein the impact was more with GT than FUM and, in addition, the combination of GT + FUM also caused significant damage to DNA of A549 and L132 cells. Thus, the genotoxic potential of GT, FUM and GT + FUM against lung epithelial cells was confirmed. Since the tail length and density reflected the extent of strand breaks in DNA, the percentage of DNA in the tail provided a quantitative measure of significant DNA damage as shown in Fig. 5b and c.

### Induction of cell cycle arrest in lung epithelial cell by GT and FUM alone and in combination

**Figure 6:**
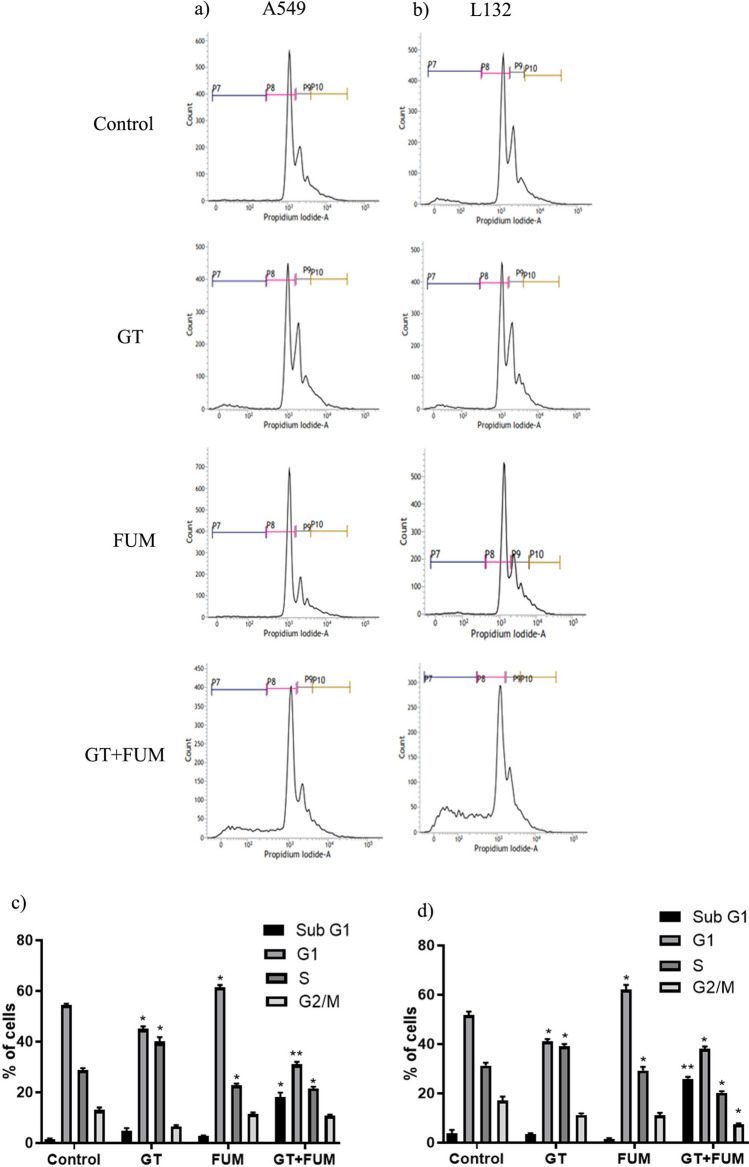
Assessment of cell cycle arrest induced by GT & FUM alone and in combination after 24 h treatment in A549 and L132 cells. (**a**, **b**) Representative contour plots of propidum iodide *vs* cell count for cell cycle distribution of A549 and L132 cells, respectively. Representation of P7, P8, P9 and P10 on the plot indicates Sub-G1, G1, S and G2/M respectively. (**c**, **d**) Bar diagram represents, percentage of cells in Sub-G, G1, S and G2/M phase of A549 and L132 cells, respectively. Data are expressed as mean ± SD of three independent experiments for each dose point (n = 48 for panels **c** and **d**). Significance between control and treatment groups was tested by adopting two way ANOVA (*p < 0.5, **p < 0.01).

The results showed that exposure of A549 and L132 cells to GT for 24 h resulted in significant increase in the proportion of cells in S-phase (30–39%) with concomitant decrease in G2/M-phase. Exposure to FUM caused significant increase of cells in G1-phase (55–63%) with concomitant decrease of cells in S-phase compared to control group. As a combination, GT + FUM produced significant decrease of cells in G1, S, G2/M phases and increase in the sub-G1 phase in both the cell lines (Fig. 6a–d).

### Mode of cell death in lung epithelial cell induced by GT and FUM alone and in combination

**Figure 7:**
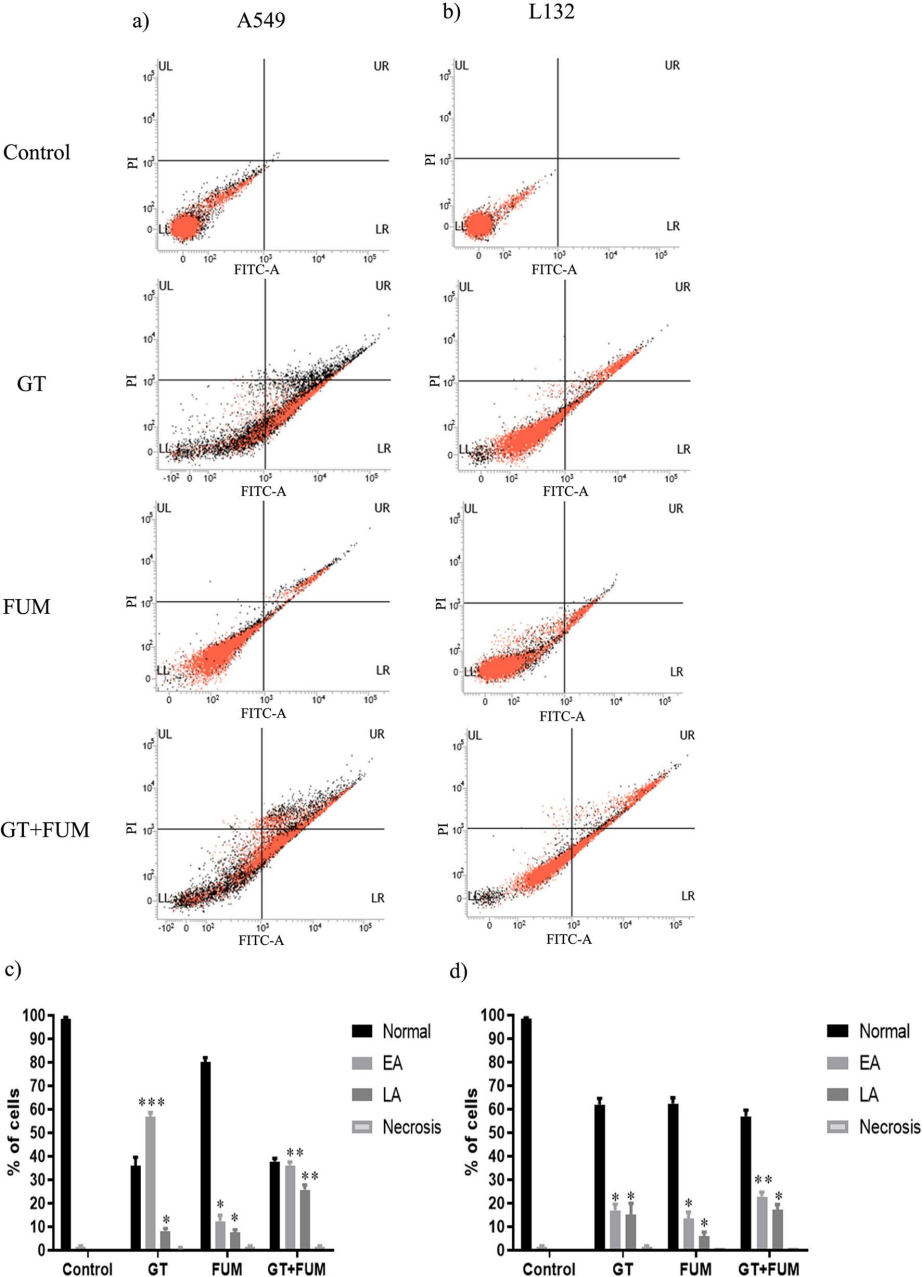
Assessment of apoptosis induced by GT & FUM alone and in combination after 24 h treatment in A549 and L132 cells. (**a**, **b**) Representative contour plots of Annexin V-FITC *vs* propidium iodide of A549 and L132 cells, respectively. Representation of LL, LR, UR and UL in the plot indicates normal, early apoptosis (EA), late apoptosis (LA) and necrosis cell populations respectively. (**c**, **d**) Bar diagram represents, percentage of normal, EA, LA and necrosis cells of A549 and L132 cells, respectively. Data are expressed as mean ± SD of three independent experiments for each dose point (n = 48 for panels **c** and **d**). Significance between control and treatment groups was tested by adopting two way ANOVA (*p < 0.5, **p < 0.01).

**Figure 8:**
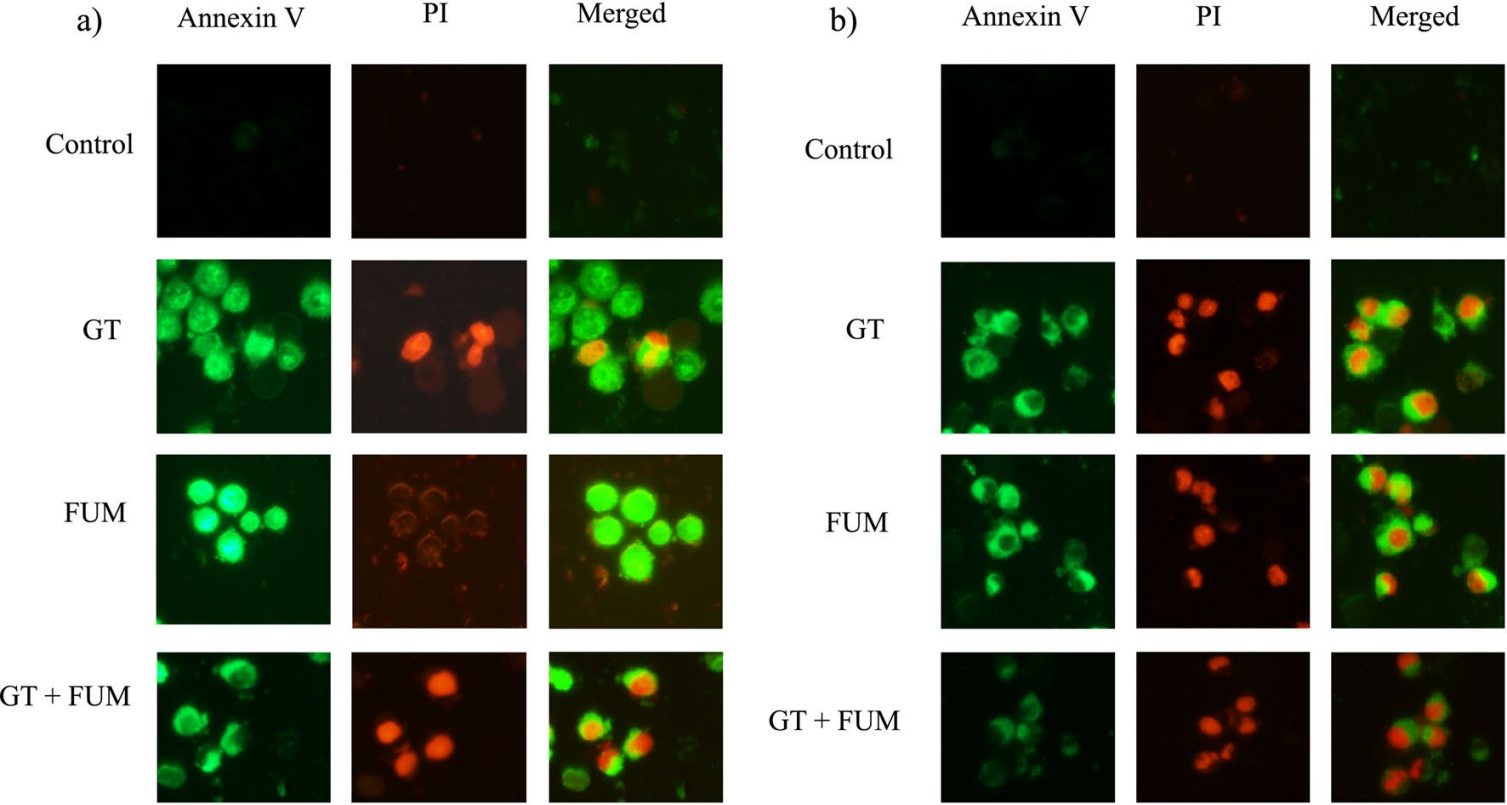
Photomicrographs showing the effect of GT & FUM alone and in combination on the mode of cell death as revealed by Annexin V/PI staining after 24 h. (**a**) Representative images of A549 cells. (**b**) Representative images of L132 cells. Normal cell are Annexin V^–^ PI^–^ (no fluorescence emission observed under FITC filter and TRITC filter), EA cells are Annexin V^+^ PI^–^ (green fluorescence observed under FITC filter and no fluorescence under TRITC filter), LA are Annexin V^+^ PI^+^ (green fluorescence observed under FITC filter and Red fluorescence under TRITC filter), Necrosis (Annexin V^–^ PI^+^ (no fluorescence under FITC filter and red fluorescence observed under TRITC filter).

Annexin V FITC-A *vs* Propidium Iodide-A plots of A549 (Fig. 7a) and L132 (Fig. 7b) cells show population of cells corresponding to viable and non-apoptotic (Annexin V^–^ PI^–^), early (Annexin V^+^ PI^–^), and late (Annexin V^+^ PI^+^) apoptotic cells. In the control (untreated) A549 (Fig. 7c) and L132 (Fig. 7d) samples, majority of cells (98%) were viable and non-apoptotic (Annexin V^–^ PI^–^). In contrast, when A549 (Fig. 7c) and L132 (Fig. 7d) cells treated with GT, FUM and GT + FUM in combination for 24 h, the percentage of Annexin V^–^ PI^–^ cells decreased and early apoptotic cell population (Annexin V^+^ PI^–^) increased significantly (Fig. 7c,d). Also, increase in Annexin V^+^ PI^+^ population was observed which indicates late apoptotic cells. In addition, GT, FUM and GT + FUM-mediated induction of apoptotic cell death was confirmed by microscopic analysis of Annexin V FITC/PI staining assay in A549 (Fig. 8a) and L132 (Fig. 8b) cells. In conclusion, GT, FUM and GT + FUM induced apoptotic mode of cell death in lung epithelial cells.

### Evidence for induction of intrinsic-pathway of apoptosis by GT and FUM alone and in combination at molecular level

**Figure 9:**
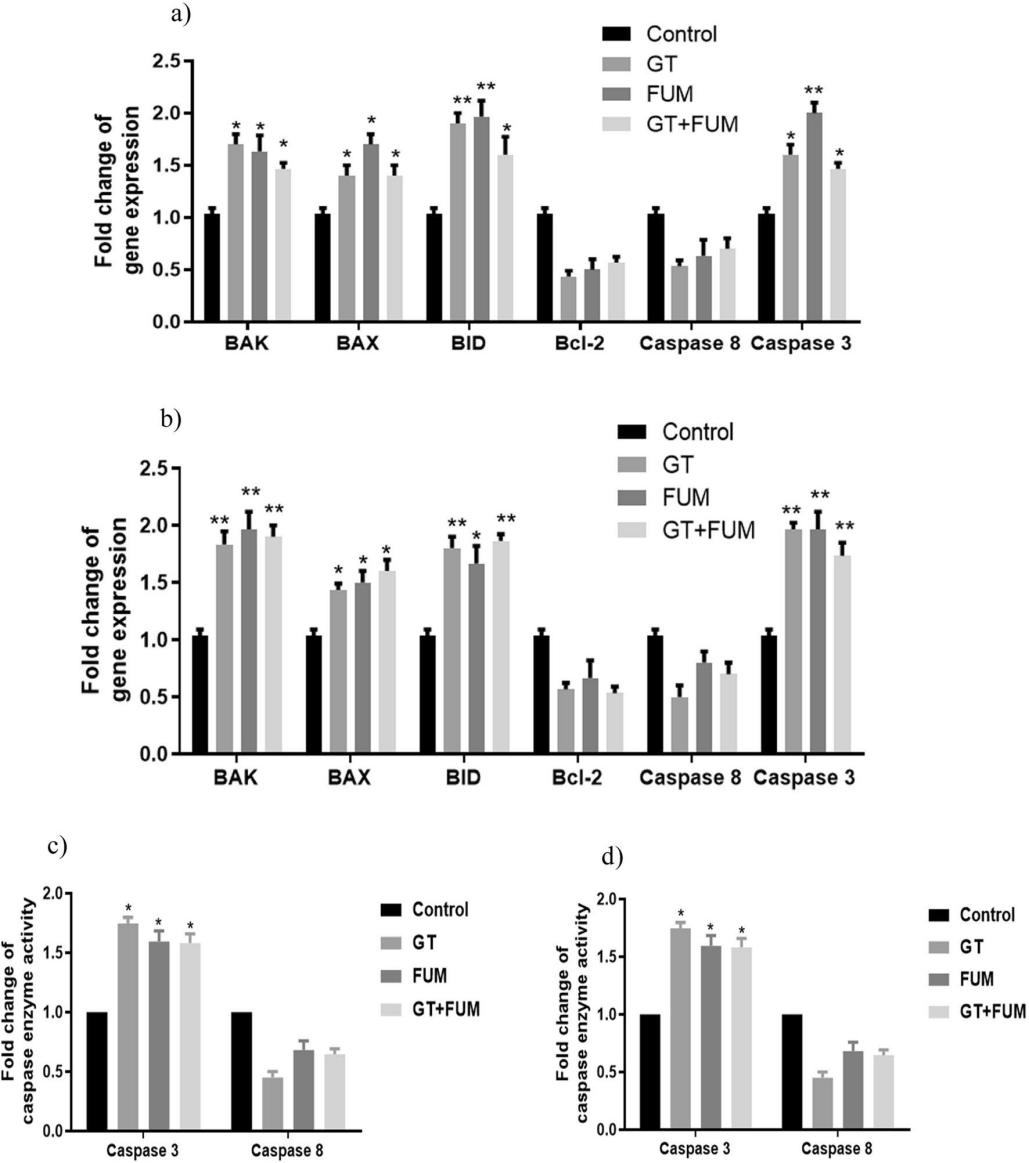
Molecular level induction of apoptosis-related genes by GT, FUM alone and in combination after 24 h. (**a**, **b**) Gene expression profile in A549 and L132 cells respectively. (**c**, **d**) Detection of caspase-3 and -8 enzyme activities in A549 and L132 cells respectively. Data are expressed as mean ± SD of three independent experiments for each dose point (n = 72 for panels **a** and **b**; n = 24 for panels **c** and **d**). Significance between control and treatment groups was tested by adopting two way ANOVA (*p < 0.5, **p < 0.01).

In order to understand the pathway of apoptosis, transcript levels of BAK, BAX, BID, Bcl-2, caspase-8 and caspase-3 were determined. As shown in Fig. 9a and b expression levels of anti-apoptotic Bcl-2 and the initiator caspase (caspase-8) of extrinsic pathway were decreased but expression levels of BAK, BAX, BID and caspase-3 were significantly increased in both A549 and L132 cells. Results of caspase 3 and 8 enzyme activity assays, shown in Fig. 9c and d, confirm that GT, FUM and GT + FUM induced the activity of caspase 3 but not caspase 8 in A549 and L132 cells. Thus, caspase activity analysis supports caspase 3 and 8 RNA expression analysis.

### Secretion of pro-inflammatory cytokines by A549 and L132 epithelial cells against GT, FUM and GT + FUM

**Figure 10:**
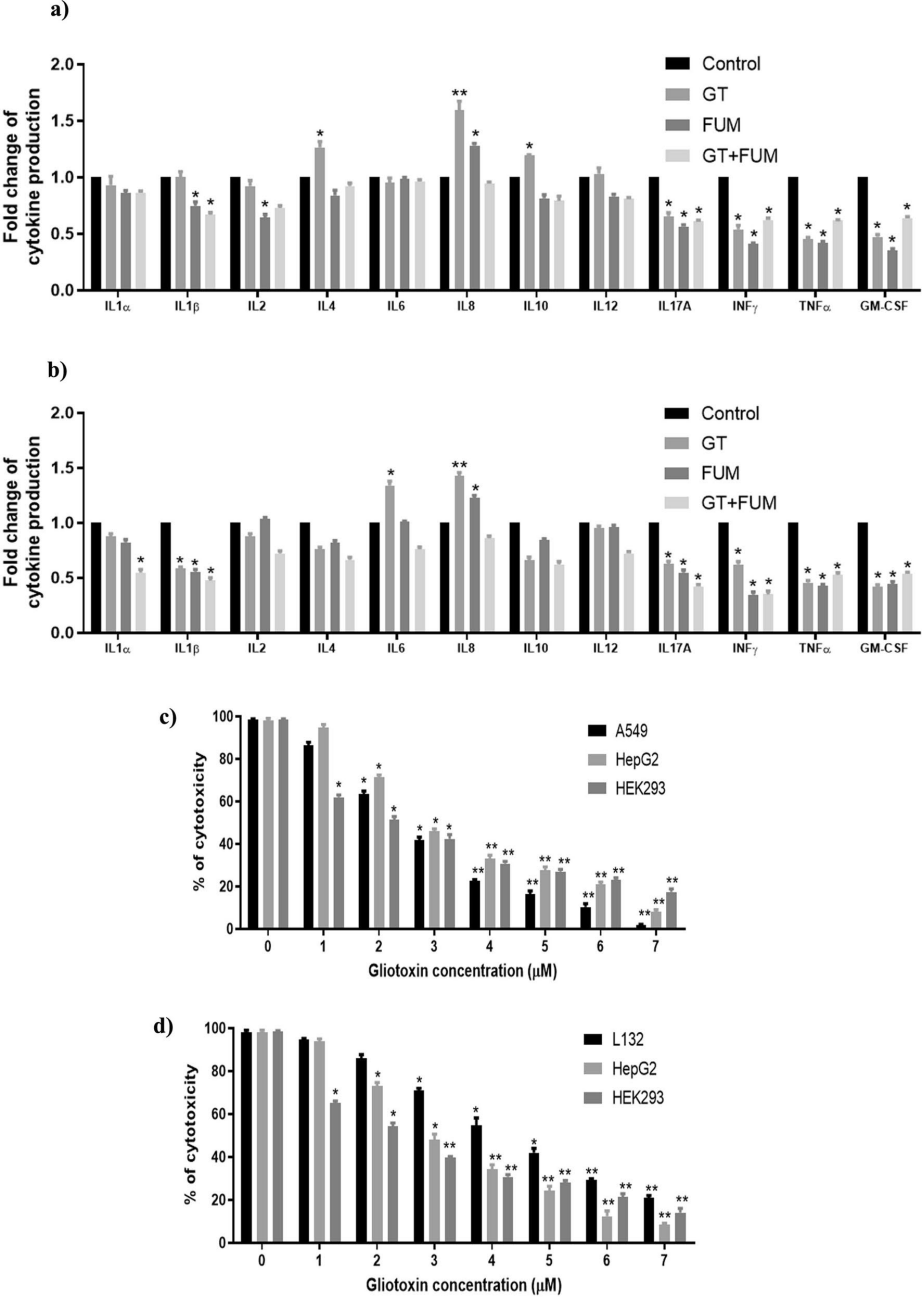
Secretion of inflammatory cytokines by epithelial cells following exposure of GT & FUM alone and in combination for 24 h and organ-specific cytotoxicity of GT adopting IdMOC technique. (**a**, **b**) Inflammatory cytokines secreted by A549 and L132 cells, respectively. (**c**) Organ-specific cytotoxicity of GT in A549 cell co-cultured with HepG2 and HEK293 cells after 24 h; (**d**) Organ-specific cytotoxicity of GT in L132 cell co-cultured with HepG2 and HEK293 cells after 24 h. Data are expressed as mean ± SD of three independent experiments for each dose point (n = 144 for panels **a** and **b**; n = 72 for panels **c** and **d**). Significance between the control and the treatment groups was tested by adopting two way ANOVA (*p < 0.5, **p < 0.01).

As shown in Fig. 10a, expression levels of IL4, IL-8 and IL-10 were significantly increased against GT whereas IL-8 alone was increased against FUM in A549 cell. In the case of L132 cell, IL-6 and IL-8 cytokine expression levels were significantly increased against GT whereas IL-8 alone was induced by FUM (Fig. 10b). As a combination GT + FUM did not induce expression of any cytokine within 24 h. Apart from up-regulation of a few cytokines as above, GT, FUM alone as well as in combination down-regulated the expressions of a few cytokines such as IL-1β, IL-17, IFN-γ, TNF-α and GM-CSF (Fig. 10a,b).

### Organ-specific cytotoxicity of GT as evidenced by IdMOC

IdMOC is a novel cell culture system that can be utilized for the evaluation of toxicity, especially for studying the comparative cytotoxicity of an agent towards multiple cell types. Therefore, in one set, A549, HepG2, and HEK293 cells in duplicates and in the other set, L132, HepG2 and HEK293 cells in duplicates were co-cultured and exposed to GT under identical conditions in the concentration range 0–7 μM for 24 h (Fig. 10c,d). The IC_50_ values for HepG2 (3 µM) and HEK293 (2.1 µM) were higher than for A549 (2.5 µM); and IC_50_ values of L132 (4.2 µM) and HepG2 (3 µM) were higher than for HEK293 (2.2 µM). The rank order of the multiple cell types towards GT cytotoxicity, from the most sensitive to the most resistant, based on the different IC_50_ values are as follows: IC_50_: HEK293 > A549 > HepG2 > L132.

## Discussion

One of the open questions in the context of IA is, how the secreted fungal mycotoxins tend to become virulent factors of *A. fumigatus* such as to damage lung epithelial cells^19^. This issue has not yet been explored with clarity, and a clear understanding can enable us to harness the defensive and offensive capabilities of *A. fumigatus* during IA. In this paper, the molecular mechanism of GT-, FUM- and GT + FUM-induced lung epithelial injury, including immune response, has been studied. Upon germination, the fungal hyphae should breach the lung alveolar surface that consists of type I and type II epithelial cells in which type I cells cover 95% of the surface and engage in gas exchange whereas type II cells cover only 5% of the surface but engage crucially in the secretion of surfactant proteins and innate immune response^2,19^. Therefore, it is important to select such cell types to study the molecular mechanism of mycotoxins to relate with IA adopting in vitro techniques. A549 is a lung carcinoma epithelial cell that represents standard model of type II cells which is capable of revealing the interaction between the pathogenic factors and the host whereas L132 is an embryonic lung epithelial cell used extensively for pulmonary toxicity studies that also manifests the phenotypes of both type I and type II alveolar cells^20,21^. Therefore, the cytotoxic potentials of GT, FUM and GT + FUM were studied in A549 and L132 cells. Results from cytotoxicity studies revealed that GT more seriously attacks epithelial cells than FUM. Previous in vitro cytotoxicity assays against A549 have also shown that GT is more cytotoxic than FUM (Table 3). Comparatively, between cell lines, lung epithelial carcinoma cell (A549) is damaged faster at lower concentrations of GT, FUM and GT + FUM than normal lung epithelial cell L132. We speculate that the cancerous nature of A549 is responsible for the change in the sensitivity towards GT and FUM. On the other hand, combination of GT and FUM at 20% and 40% of the respective IC_50_ concentrations acting on A549 and L132 cells led to increase in cytotoxicity compared to individual toxins at their respective IC_50_ concentrations.

**Table 3:** Summary of cytotoxicity studies on GT and FUM against A549 cells.

Cell type	IC_50_ value/time/assay	Toxic effect	Reference
**GT**			
A549	0.1 µg/ml (0.3 µM) /24 h/neutral red assay	Cytotoxicity	^22^
A549	0.16 µg/ml (0.4 µM)/48 h/MTT assay	Cytotoxicity	^23^
A549	0.16 µg/ml (0.0005 mM)/24 h/MTT assay	Cytotoxicity	^24^
A549	0.04 µg/ml (0.12 µM)/not mentioned/MTT	Cytotoxicity	^25^
A549	0.097 µg/ml (0.3 µM)/24 h/MTT assay	Cytotoxicity	^26^
**FUM**			
A549	16.5 µg/ml (35.9 µM)/24 h/neutral red assay	Cytotoxicity	^22^
A549	3.2 µg/ml (6.97 µM)/not mentioned/MTT assay	Cytotoxicity	^25^

Though the clinical presentation of IA is relatively rapid, ranging from days to a few weeks for neutropenic host, non-neutropenic patients such as solid organ transplant recipients (particularly lung and heart–lung transplant recipients), patients with AIDS, COPD, and the critically ill intensive care unit (ICU) patients are relatively indolent and the condition progresses over weeks rather than over days (up to 12 weeks)^27–29^. Therefore, total net immunosuppression of the host usually reflects the progression of fungal spread in lungs during the course of IA^27–29^. Thus, the concentration of mycotoxins produced by *A. fumigatus* may also vary between host’s immune surveillances. In addition, production of mycotoxins may vary between species and even within a given species^30^. Hence, it is nearly impossible to speculate conclusively about fungi producing a toxin at a particular concentration under certain conditions. At least any such statement must remain subject to severe uncertainties^30^.

However, there are a few in vitro reports to endorse the concentration of GT and FUM produced by *A. fumigatus* in which, clinical strains of *A. fumigatus* at 1 × 10^5^ cfu can produce GT at the maximum of 21.35 µg/mL and mean of 5.75 µg/mL in RPMI 1,640 medium at 37°C^9^ and environmental strains of *A. fumigatus* at 9.3 × 10^6^ cfu can produce 365 µg/mL of FUM in YES medium at 25 °C^22^. These concentrations reveal toxin producing capacity of *A. fumigatus* under favorable conditions but this may or may not be realistic in true in vivo pathophysiological condition. In addition, we can at least understand the diagnostic concentrations of GT from the Table 1 but, for FUM, no such measure is available so far to directly compare the in vivo concentrations with the studied concentrations. Therefore, our study was intended to find the half maximal cytotoxic concentrations (IC_50_) of GT, FUM and GT + FUM instead of solely relying on particular concentrations at in vivo conditions (Table 1). Therefore, our study shows that at a defined concentration GT and FUM alone and in combination can act synergistically to induce epithelial damage to lungs.

In general, mycotoxins use ROS as a weapon to alter the cellular redox signaling to initiate destructive signals against cell endurance^6^. In order to counteract the imbalance in redox state, the host’s body would kick off stress-related or antioxidant gene expressions to maintain cellular homeostasis^31^. As hypothesized, we found GT, FUM and GT + FUM to significantly induce cellular level of ROS in lung epithelial cells within 24 h and also concurrent increase in the gene expression of FOXO1, which is an important transcription factor that regulates cellular stress response and promotes cellular antioxidant defense systems such as catalase, SOD1, GSTP1, GR, GPx1 and GPx2 within 24 h in both A549 and L132 cells. Although antioxidant enzyme expressions are increased, adequate changes to reduce the level of generated ROS are not evidenced in Fig. 2b and c. To conclude further about the action of GT, FUM and GT + FUM on cellular antioxidant system, protein level enzyme activities of antioxidants are required to be studied.

Further, the elevated level of ROS guided us to find the healthy state of mitochondria that play a vital role in the sudden burst of ROS upon treatment of mycotoxins. We found an increase in mitochondrial membrane depolarization in both A549 and L132 cells by GT, FUM and GT + FUM. This shows the possibility that collapse of mitochondrial membrane is mediated by ROS or the production of ROS is followed by mitochondrial membrane depolarization. This comprehension is supported by the concept “Reactive Oxygen Species (ROS)-induced ROS-release” (RIRR) in which the excessive ROS can collapse mitochondrial membrane that would lead to temporary increase in ROS generation by the electron transfer chain^32^. Later, ROS is released to cytoplasm to inflict damage to the neighboring mitochondria. This mitochondrion-to-mitochondrion ROS-signaling signifies positive signals for better ROS production which would lead to momentous mitochondrial and cellular injuries^32^.

Further, the singlet oxygen of ROS can readily react with proteins, DNA and lipid molecules of the cell. Thus, oxidative stress/pathological conditions of the cell can build unfolded proteins to accumulate in the lumen of the ER, which we studied indirectly by means of GRP78 and HSP70 expression analyses. Both GRP78 and Hsp70 are chaperons that are up-regulated inside the cells to increase the folding capacity of unfolded proteins and to assist a wide range of folding processes, including refolding of mis-folded and aggregated proteins in the ER, respectively^33,34^. The increase in GRP78 and Hsp70 that we found confirms that the ROS that is generated reacts with protein molecules and also alters the post-translational process that occurs inside the lumen of ER after treating the cells with GT, FUM and GT + FUM. Previous in vivo and in vitro studies have demonstrated an increase in ER stress markers in murine lungs and tracheal epithelial cells challenged with *A. fumigatus* antigens^35^. We found genotoxic potential of GT and FUM against the epithelial cell lines A549 and L132, evidencing single strand DNA breaks. These results are consistent with the earlier studies on genotoxicity of GT and FUM^36,37^. In addition, the combination of GT + FUM at low concentrations showed DNA-damaging potential which is threatening compared to individual mycotoxins at corresponding concentrations. Though the protagonist of the entire event caused by the test mycotoxins is putatively identified as ROS, direct DNA damage is also likely to happen. Further investigations using strong antioxidants are needed to find how precisely GT, FUM and GT + FUM-treatments cause DNA damage. Further, causation of S and G1 phase arrest by these cytolethal mycotoxins, GT, FUM and GT + FUM, during cell cycle is evidenced. Looking into the intricate mechanism of action, the data indicate that both GT and FUM enter the cell cytoplasm to bring about cell cycle arrest. Thereupon, GT induces cell cycle arrest by interfering with the DNA doubling S-phase through imminent DNA damage. This is followed by induction of cell cycle arrest by FUM at G1 phase, indicating that it interferes with the transcriptional and post-transcriptional events that are crucial for the G1 to S-phase transition. The difference in the outcomes of previous studies on GT-induced effect on cell cycle analysis and our study may be due the difference in concentrations^23,38^ whereas the outcomes of cell cycle analysis in respect of FUM are similar^39^. The GT + FUM combination produced significant decrease of A549 and L132 cells in S, G2, and M phases and increase of cells in sub-G1 phase, which point to inhibition of mitosis and induction of apoptosis. This indicates that GT + FUM induce DNA damage but the cells which are not able to repair the damage succumb to apoptosis. These results support our data on genotoxic potential of GT, FUM and GT + FUM against A549 and L132 cells.

Apparently, intracellular cues such as increased cytotoxicity, ROS generation, stress signaling pathways, mitochondrial membrane damage and cell cycle arrest can lead to cell death. The results of Annexin V/PI flow cytometry confirmed that both GT and FUM alone as well as in combination induced apoptosis rather than necrosis in both A549 and L132 epithelial cells. Our results are consistent with previous reports in respect of GT and FUM^23,24,40^. Apoptosis can be initiated by one of two pathways, caspase-8-associated death receptor pathway, i.e. extrinsic pathway, and mitochondria-mediated intrinsic pathway. Binding of ligands with death receptors will activate a series of downstream factors, including caspase-8 which is a critical mediator of the extrinsic pathway. Intrinsic pathway of apoptosis is initiated via mitochondrial membrane pore formation where the stress signals cause the binding of cytoplasmic proteins BAX and BID with mitochondrial membrane protein BAK. At the culmination of both of these apoptotic pathways, initiation of executioner caspase 3-dependent proteolytic cascade is considered a key factor of apoptotic cell death^6^. Our expression analysis revealed decrease of anti-apoptotic Bcl-2 protein and increase of pro-apoptotic BAX, BID and BAK, validating the participation of mitochondria in the cell death. Decrease in the RNA expression/activity of caspase 8 and increase in the expression/activity of caspase 3 authenticate that caspase-dependent mitochondria-mediated intrinsic pathway of apoptosis is induced by GT, FUM and GT + FUM in lung epithelial cells. Our results with regard to apoptosis are consistent with previous findings in respect of GT and FUM^41,42^.

Though we found generation of ROS to be a crucial event in the molecular mechanism of cell death induced by GT, FUM and GT + FUM, we still have question on the production of intracellular ROS in response to GT and FUM. Previous studies let us believe that the structure of GT can exist in either disulfide or dithiol forms, depending on the reducing or oxidizing environment^43,44^. Further, GT exists naturally in an oxidized disulphide form but upon cellular uptake GT is chemically reduced to the dithiol form by intracellular glutathione (GSH). Thus, GT is increasingly accumulated in a cell in a glutathione-dependent manner^43,44^. However, induction of ROS followed by apoptosis is observed in many cell lines because reduced-GT can be oxidized to GT, thus, releasing ROS, and it can also form mixed disulfides by thiol groups with proteins or antioxidants such as glutathione^45,46^. Then, oxidation of the intracellular toxin results in rapid efflux from the cell which also occurs when glutathione levels decrease following induction of apoptotic cell death by the toxin^43,44^. Further, reduced dithiol form of GT has the ability to chelate Zn^2+^ ions from intracellular metalloenzymes and target thioredoxin redox system^47,48^. Also, GT is involved in activation of phospholipase D which catalyzes hydrolysis to produce the signal molecule phosphatidic acid, soluble choline and actin cytoskeleton rearrangement^49,50^. On the other hand in case of FUM, total glutathione content is significantly higher in cell lines insensitive (human umbilical vein endothelial cells) to FUM than in sensitive cell lines (human colon cancer cell line)^51^. Also, FUM has been demonstrated to affect not only methionine aminopeptidases proteins (family of intracellular proteolytic enzymes) but also all the subsequent activities necessary for the effective functioning of proteins related to cell viability and growth^41^. These studies clearly show that glutathione, the hall mark of cellular redox homeostasis, is affected by both GT and FUM, and the structural advantages of theses mycotoxins could be the reason for the generation of intracellular ROS and thus affect several intracellular proteins and induce apoptosis. Thus, GT and FUM in combination can act synergistically against cellular glutathione redox system.

Further, lung epithelial cells can initiate an inflammatory response against mycotoxins to disrupt fungal growth. Previous studies on immunological responses either for *A. fumigatus* or culture filtrate of *A. fumigatus* showed increase of cytokines IL-4, IL-10, IL-6, IL-8, IFN-g and TNF-α^1,52^. Although, it is important to find stimulatory effect of GT, FUM and GT + FUM involved in the inflammation of epithelial cells which act as a first-line defense signals that release pro-inflammatory cytokines to attract neutrophils, macrophages and dendritic cells at the site of infection. In this way we project the mycotoxins to act as virulence factors without any fungal antigen moiety to provoke innate immune response of host epithelial cells rather as a physical barrier of lung. As revealed in the results, IL-8 is the common pro-inflammatory signal produced by both A549 and L132 cells against GT and FUM. IL-8 is a chemokine that contributes to the effective recruitment and activation of neutrophils at the site of infection. This clearly shows that both type 1 and type 2 epithelial cells enable neutrophil-mediated-inflammatory responses against GT and FUM. These results are consistent with previous studies showing production of IL-8 upon conidial germination and hyphal growth of *A. fumigatus* inside epithelial cells. Apart from IL-8, GT induced IL-4 and IL-10 in A549 cell. T helper 1 (Th1) anti-inflammatory cytokines IL-4 and IL-10 are involved in the maintenance of homeostasis and, in particular, in controlling pro- and anti-inflammatory cytokines involved in infectious, allergic and autoimmune diseases. In addition, these anti-inflammatory cytokines limit the immune response to self- and non-T-self antigens, protecting the host from excessive Th1 pathology and preventing tissue damage^1^. Further, previous reports have shown that immune response initiated by IL-4 and IL-10 activates T helper 2 (Th2) cell differentiation and inhibits Th1 and Th17 response. IL-4 and IL-10 deficient mice show lower *A. fumigatus* burden and increased survival rates compared to wild type mouse in invasive pulmonary aspergillosis^1^. Thus, we speculate that exposure of GT would even enhance neutrophil recruitment at the site of infection to cause neutrophil-mediated inflammatory tissue damage and, thus, IL-4 and IL-10 cytokines are increased by epithelial cells to prevent the excessive inflammatory response of Th1 to avoid self-tissue damage. On the other hand, in L132 cell, production of IL-6 cytokine was also significant apart from IL-8. The IL-6 is an important proinflammatory cytokine involved in regulation of Th17 response during *A. fumigatus* infection. Further, Th17cells activate neutrophil migration towards the infected area and thereby increase inflammation. Over all, the results clearly show that recruitment of neutrophil to the site of infection could be the innate immune response of epithelial cells against the fungal mycotoxins GT and FUM. Moreover, these toxins have been proved for their ability to inhibit the function of neutrophils. This could be one of the reasons for neutropenia patients to manifest frequent IA infection due to fewer neutrophils to fight the pathogen^27–29^. An earlier study has shown similar results in LPS induced A549 cells against GT^46^. In addition, the combination of GT + FUM at a lower concentration could not induce any cytokine expression within 24 h which is perhaps a good strategy to bring about epithelial cell death without inflammatory response or may take more than 24 h to alter the immunological response. This brings up the dangerous effect of GT + FUM as a combination to damage epithelial cells invasively, without any resistance from the host immune system. However, the molecular mechanisms by which these toxins induce cytokine expression remains to be studied. Further, in vivo investigation about immune regulation mechanism might reveal some potential targets for diagnosis and treatment of pulmonary fungal diseases.

Once the epithelial layer of alveoli is damaged, *A. fumigatus* arrives at the endothelium of the blood vessel to become an angiotrophic fungus and, thus, the disease becomes invasive and spreads to other organs. Disseminated aspergillosis is defined as the involvement of at least two non-contiguous organ sites. *Aspergillus* may disseminate from lungs to invariably every organ^28^. Disseminated disease has been described in 9–36% of kidney recipients, 15–20% of lung recipients, 20–35% of heart recipients and 50–60% of liver recipients with IA^28^. Therefore, we followed up with study of multiple organ toxicity of GT, adopting IdMOC, a novel cell culture system that is appropriate in the context of evaluation of comparative cytotoxicity of an agent towards multiple cell types. IA is the most severe form of disease that occurs when the infection spreads rapidly from lungs to liver, heart, kidneys or skin. So far, GT is the only mycotoxin that has been identified in the sera of IA patients, and considered as a virulent factor of *A. fumigatus.* Thus, the presence of GT in the sera of IA patients justifies evaluation of multiple organ toxicity or organ-specific toxicity. As shown in the results, A549 (representing lung epithelial cell) and HEK (representing renal epithelial cell) are likely to be more sensitive and liable for GT-induced cytotoxicity than HepG2 (representing liver epithelial cells) and L132 (representing both types 1 and II lung epithelial cells) which are fairly sensitive. Studies on potential toxicokinetic variations between cell models can explain the sensitivity issues. Also, future studies that associate neuronal cells and skin cells are required to relate the clinical symptoms of brain and skin damage in IA patients^28^.

**Figure 11:**
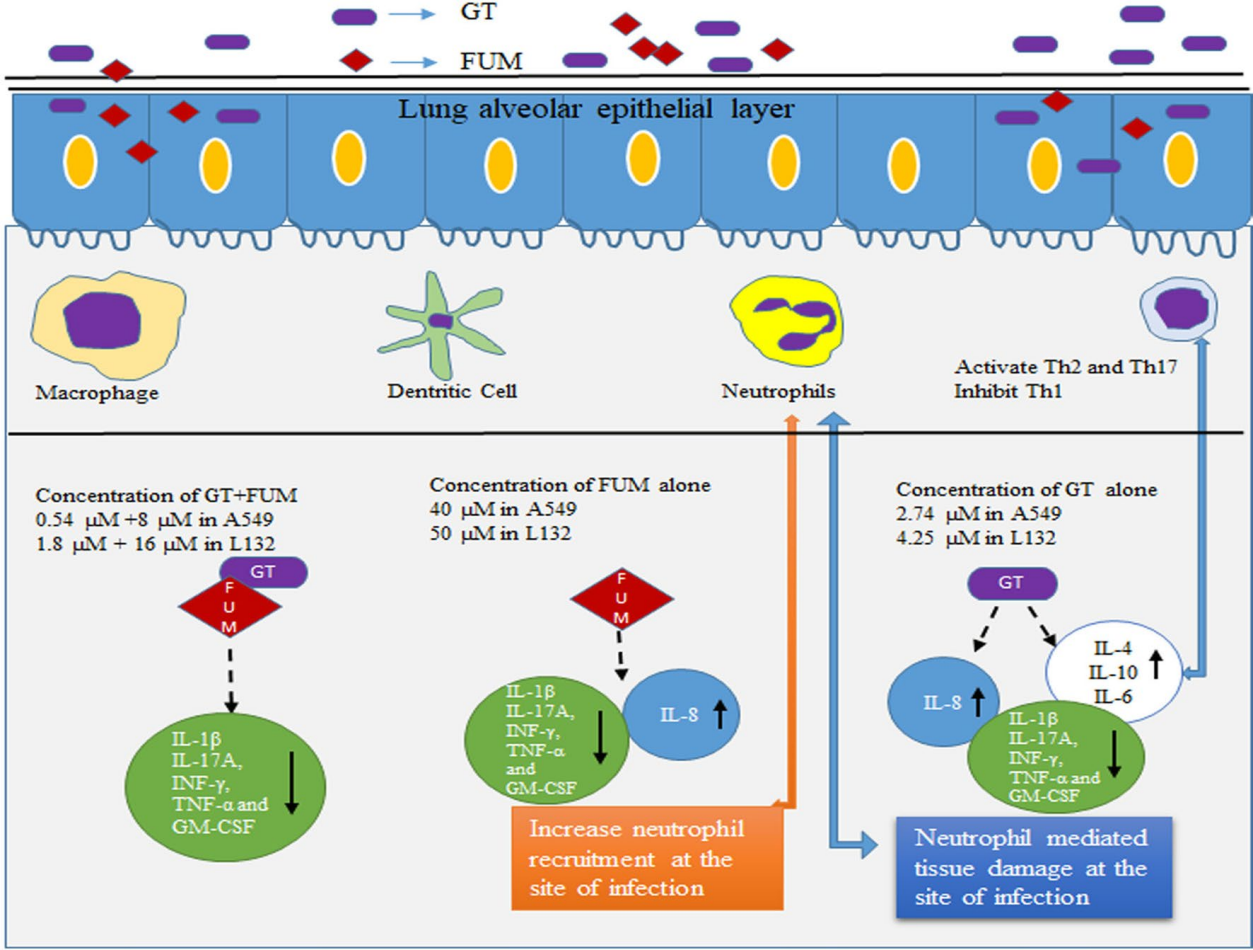
Represents how GT, FUM and GT + FUM act as virulent factors to provoke/evade immune surveillance of host without any fungal antigen.

In conclusion, our study shows that at a specific concentration GT and FUM interact synergistically to induce cytotoxicity in lung epithelial cells. The molecular mechanism of toxicity of GT, FUM and GT + FUM to the lung epithelial cells greatly depends on ROS-mediated, ER-stress-induced, mitochondria-mediated, caspase-dependent intrinsic pathway of apoptosis. ROS are produced after mitochondrial damage and, thus, mitochondria are both the source of ROS and target of ROS. GT-, FUM- and GT + FUM-induced DNA damage is mediated either by an ROS-dependent course or by a direct attack. In addition, GT, FUM and GT + FUM may induce unfolded protein accumulation. Although there are signs of indication that GT and FUM can induce neutrophil recruitment at the site of infection as revealed in the expression of cytokines in epithelial cells (Fig. 11) and GT would damage epithelial cells by neutrophil-mediated inflammatory response, further studies are needed to confirm the hypothesis made out in the study. GT is identified to be highly cytotoxic to renal epithelial cells at low concentration and type II epithelial cells at high concentration. It is already known in an in vivo model that GT is a virulence factor and FUM is a likely factor in cellular damage during infection of the host by *A. fumigatus*^14,53^. This is further substantiated by the down-regulation of cytokines that are generally involved in the regulation of immune system as well as in the control of the fungal pathogenesis. Taken together, these findings indicate that GT and FUM alone and in combination are capable of damaging lung epithelial cells and, thus, these mycotoxins could be an attractive target for drug development for IA.

## Materials and methods

### Materials

GT (CAS Number 67-99-2; ≥ 98% purity) and FUM (CAS Number 23110-15-8; ≥ 90% purity) were obtained from Sigma Aldrich Chemical Company (St. Louis, MO, USA), DMEM, Phosphate-Buffered Saline (PBS), trypsin–EDTA, penicillin and streptomycin were obtained from Hyclone laboratories (South Logan, Utah). F etal bovine serum (FBS) was obtained from Gibco (UK). 3-(4,5-dimethylthiazol-2-yl)-2,5-diphenyltetrazolium bromide (MTT), AnnexinV, Propidium Iodide (PI) and RNase I were obtained from Sigma Aldrich Chemical Company (St. Louis, MO, USA). All other chemicals and reagents were of analytical grade.

## Methods

### Cell culture

Human lung epithelial carcinoma cell A549, normal lung epithelial cell L132, human hepatocarcinoma cell HepG2 and human embryonic kidney cell HEK293 were obtained from National Centre for Cell Science (NCCS), Pune, India. The cells were maintained in DMEM medium supplemented with 10% FBS, and with 100 U/mL each of penicillin and streptomycin as antibiotics in a humidified atmosphere of 5% CO_2_ and 95% air, in a CO_2_ incubator (Eppendorf, India).

### Integrated discrete multiple organ co-culture (IdMOC)

The IdMOC 96 well plates were gift from Dr. Albert P. Li, President and CEO of In Vitro ADMET Laboratories LLC, Columbia, USA. The concepts and applications of IdMOC have been reviewed^54^.

### Cytotoxicity assay

Colorimetric cytotoxicity assay was performed according to Mosmann et al.^55^ A549 and L132 cells were seeded in 96 well plate at 5 × 10^3^ cells/well and treated with different concentrations of GT, FUM and combination of the two, dissolved in dimethyl sulfoxide (DMSO) for 24 h, at 37 °C. DMSO was used as the solvent control. After 24 h incubation, 20 μL of MTT [3-(4,5-dimethylthiazol-2-yl)-2,5-diphenyltetrazolium bromide] solution (5 mg/mL in PBS) was added to each well, and incubated for 3 h at 37 °C. The medium was then removed and the purple formazan product was dissolved in 100 μL of DMSO. The absorbance was measured at 570 nm (measurement) and 630 nm (reference) using a 96-well plate EnSpire multimode reader (Perkin Elmer, Waltham, MA, USA). Data were collected for triplicates and used to calculate the respective means and the standard deviations. The percentage viability (*visa vi* cytotoxicity) was calculated from this data using the following formula:

$$Percentage \, \;viability \, \left( {visa \, vi,\; \, cytotoxicity} \right) \, = \, Mean\; \, OD\; \, of\; \, untreated \, \;\;cells \, \left( {control} \right) \, - \, Mean\; \, OD \, \;of\; \, treated \, \;cells/Mean \, \;OD \, \;of \, \;untreated \, \;cells \, \left( {control} \right) \, \times \, 100.$$

From the values thus obtained, the IC_50_ was calculated. IC_50_ is defined as concentration of the test substance at which cell viability is decreased to 50%, meaning 50% cells are dead.

### Isobologram method of toxicity assessment of binary combination of mycotoxins

The types of interaction of GT and FUM mixtures were determined by isobologram analysis using CompuSyn software (version 1.0.1 [2004]; CompuSyn, Inc.) established by Chou and Talalay^56^. In this analysis, combination index (CI) is a quantitative parameter used to evaluate the types of interaction and the corresponding influential level among binary or more compounds. CI < 1 indicates synergism, CI = 1 indicates additive effect and > 1 indicates antagonism of the compounds (in the present case, mycotoxin combinations).

### Reactive oxygen species (ROS) assay for the assessment of oxidative stress

The generation of intracellular ROS was measured using the fluorescent probe 2′,7′-dichlorofluorescein diacetate (DCFH-DA) according to Wang and Joseph^57^. To evaluate mycotoxins-induced DCF oxidation, 1 × 10^6^ counts of A549 and L132 cells, as the case may be, were seeded in each of the 6 wells of six well plates and treated with the positive control (H_2_O_2_) and IC_50_ concentration of GT and FUM alone and the two in combination at 20% and 40% of the respective IC_50_ values, for 6 h, 12 h and 24 h against A549 and L132 cells, respectively. After that, the wells were loaded with 5 μM of DCFH-DA and incubated for 30 min at 37 °C. ROS generation was measured in EnSpire multimode reader (Perkin Elmer, Waltham, MA, USA) at an excitation wavelength of 485 nm and emission wavelength of 520 nm. Results are expressed as the ratio mycotoxin-induced DCF fluorescence/control fluorescence.

### Single-cell gel electrophoresis (comet assay) for genotoxicty assessment

DNA damage was detected adopting the comet assay^58^. The technique combines DNA gel electrophoresis with florescent microscopy to visualize migration of DNA from individual agarose-embedded cells. The cells were treated with the IC_50_ concentration of GT and FUM, alone and in combination at 20% and 40% of the respective IC_50_ values, for 24 h against A549 and L132 cells, respectively. The harvested cells were suspended in low melting point agarose in PBS and pipetted out to microscope slides pre-coated with a layer of normal melting point agarose. The slides were immersed in pre-chilled lysis buffer (2.5 M NaCl, 100 mM Na_2_EDTA, 10 mM Tris, 0.2 mM NaOH [pH 10], and Triton X-100) and incubated overnight at 4 °C in order to lyse the cells and to permit DNA unwinding. Thereafter, the slides were exposed to alkaline buffer (300 mM NaOH, 1 mM Na_2_-EDTA, [pH > 13]) for 20 min at 20 V to allow DNA unwinding. The slides were washed with buffer (0.4 M Tris, pH 7.5) to neutralize excess alkali before staining with EB. The EB-stained slides were observed in a fluorescent microscope (Nikon, Japan). One hundred and fifty cells, in triplicate, from each treatment group were digitized and analyzed using CASP software. The extent of DNA damage was assessed from the shape and area of the comet tail. The images were used to determine the DNA content of individual nuclei and to evaluate the degree of DNA damage representing the fraction of total DNA in the tail.

### JC1 staining for assessment of mitochondrial trans-membrane potential

Mitochondrial trans-membrane potential (ΔΨm) was measured using the fluorescent probe JC-1 (5,5′,6,6′-Tetrachloro-1,1′,3,3′-tetraethyl-imidacarbocyanine iodide) by adopting the method of Reers et al.^59^. The cells were grown in glass cover slips placed in the wells of 6-well plates and treated with IC_50_ concentrations of GT and FUM alone and the two in combination at 20% and 40% of the respective IC_50_ values, for 24 h, against A549 and L132 cells. The cells were stained with JC-1 dye after 24 h exposure. The mitochondrial depolarization patterns of the cells were observed in the fluorescent microscope (Nikon, Japan) and the fluorescence pattern of cells (red, normal; green, mitochondrial membrane depolarized) were observed and recorded.

### Flow cytometry analysis for cell cycle arrest

Cell cycle arrest was detected using flow cytometry^60^. The cells were treated with the IC_50_ concentrations of GT and FUM alone and in combination at 20% and 40% of the respective IC_50_ concentrations, for 24 h, against A549 and L132 cells. The cells were harvested, washed with PBS, centrifuged at 1,000 rpm for 5 min, and fixed with 1 mL 70% ice cold ethanol overnight at 4 °C. Later, the ethanol was removed and 10 µL of RNase A (10 mg/mL) was added and incubated for 30 min. Again, cells were washed with PBS and re-suspended in 1 mL PBS with 50 µL Propidium Iodide (1 mg/mL) for 30 min in dark. The cells were analyzed using the BD FACSverse flow cytometer (Becton–Dickinson), and a peak fluorescence gate was used to discriminate the aggregates. A total of 20,000 events were acquired and three replicates were used per experimental condition. The percentage of cells in each phase was determined in reference to untreated cells.

### Flow cytometry analysis of apoptosis

Detection of apoptosis by flow cytometry was conducted using the protocol given in manufacturer’s instruction (BD Biosciences, cat no: 556547). The cells were treated with the IC_50_ concentration of GT and FUM alone and in combination at 20% and 40% of the respective IC_50_ values, for 24 h against A549 and L132 cells. The cells were harvested, washed with PBS, centrifuged at 1,000 rpm for 5 min and re-suspended in100 µL binding buffer. Five microliters of Annexin-V and Propidium Iodide were added and incubated for 15 min at room temperature in dark. The stained cells were measured at 515 nm fluorescence emission using FACSverse flow cytometer (BD) and the data were processed by FACSverse software.

### Annexin V-FITC/PI binding assay for the assessment of cell death

Annexin V-FITC/PI staining was performed to assess the morphological characteristics of cell death by apoptosis or necrosis^61^. The A549 and L132 cells were cultured in 6-well plates and treated with IC_50_ concentrations of GT and FUM alone and in combination at 20% and 40% of the respective IC_50_ values, for 24 h, respectively. The treated and untreated cells were centrifuged at 3,000 rpm for 5 min, incubated with Annexin V-FITC/PI and examined under FITC filter for green emission of Annexin V-FITC and TRITC filter for red emission of PI using an inverted fluorescent microscope (Nikon, Japan).

### Real time PCR for gene expression analysis

Total RNA were isolated from untreated and treated A549 and L132 cells (2 × 10^6^ in T_25_ culture flask) using the TRIzol Reagent (Invitrogen; USA) and quantified using a NanoDrop spectrophotometer (Bio Drop Duo, UK). First strand cDNA was synthesized with 1 μg of RNA and oligo(dTs) to a final volume of 20 μL according to the manufacturer’s instruction (Thermo Scientific, USA). qPCR was performed as 10 μL reaction mix with a final concentration of 1 × SYBR Green (Roche), 0.5 μL of each primer, and the cDNA that was synthesized in the earlier step. To detect non-specific amplification, negative controls (no template control and minus RT control (only with RNA) were included in the assays. The amplicons were sequenced to confirm their identity**.** The reactions were carried out using a single step real time PCR machine (Roche, USA) under the following conditions: initial denaturation at 95 °C for 5 min, followed by 30 cycles of 3 step amplification at 95 °C for 10 s, 50/60 °C for 30 s and 72 °C for 10 s.

Primers for the apoptosis and stress-related genes were selected from the earlier reports^62–66^. GAPDH served as the internal control to normalize the RNA level. Three technical repeats and experimental replicates were performed for each gene.

### Caspase-3 & -8 activity assay for apoptotic cell death

Caspase-3 and -8 activities were determined using assay kit, according to the manufacturer's instructions (Cat. No. K106-100 & K113-25 Biovision, Inc., Milpitas, CA, USA). A549 and L132 cells were cultured in 6-well plates and treated with IC_50_ concentrations of GT and FUM alone and in combination at 20% and 40% of the respective IC_50_ values, for 24 h. The control and treated cells were lysed in sample lysis buffer (Biovision, Inc.). The homogenates were then centrifuged at 10,000×*g* and 4 °C for 10 min and the supernatant was collected for protein determination. The cell lysates were then exposed to DEVD-pNA and IETD-pNA substrate conjugate provided in the kit for caspase-3 and -8, respectively, for 1 h at 37 °C. The sample was measured at 400–405 nm in multimode plate reader (Model: Enspire, Perkin Elmer, CA, USA).

### Multi-analyte ELISA array for detection of inflammatory cytokines

The Human Inflammatory Cytokines Multi-Analyte ELISArray kit (Cat. No. MEH-004A; Qiagen, Valencia, CA, USA) was used to detect a panel of 12 cytokines according to the manufacturer’s protocol. The ELISA panel included: Interleukin (IL)-1α, IL-1β, IL-2, IL-4, IL-6, IL-8, IL-10, IL-12, IL-17A, interferon-γ (IFN-γ), tumor necrosis factor-α (TNF-α) and granulocyte–macrophage colony-stimulating factor (GM-CSF)^67^.

### Integrated discrete multiple organ toxicity assay using IdMOC technology for organ-specific toxicity

IdMOC 96 well plates with 16 containing wells, each with 6 inner wells, were used to discretely co-culture A549, HepG2, and HEK293 cells (Plate 1) and L132, HepG2, and HEK293 (Plate 2). In each inner well 10,000 cells were plated in 80 μL of the medium specific for each cell type. The cells were cultured in an incubator kept at 37 °C, and a highly humidified atmosphere of 95% air and 5% carbon dioxide for 24 h before treatment. On the day of treatment, the medium was removed from all inner wells of the IdMOC plate and replaced with 2.5 mL DMEM containing various concentrations of GT. Treatment was performed in triplicate for an incubation period of 24 h. After 24 h, 50 μL of MTT [3-(4,5-dimethylthiazol-2-yl)-2,5-diphenyltetrazolium bromide] solution (5 mg/mL in PBS) was added to each well, and incubated for 3 h at 37 °C. The medium was removed and the purple formazan product was dissolved in 50 μL of DMSO. The absorbance was measured at 570 nm (measurement) and 630 nm (reference) using a 96-well plate EnSpire multimode reader (Perkin Elmer, Waltham, MA, USA)^7^. Data were collected for triplicates and used to calculate the respective means and the standard deviations. The percentage viability (*visa vi* cytotoxicity) was calculated from this data using the following formula:

$$Percentage \, \;of\; \, viability \, \left( {visa\; \, vi, \, \;cytotoxicity} \right) \, = \, Mean \, \;OD \, \;of \, \;untreated \, \;cells \, \left( {control} \right) \, - \, Mean\; \, OD\; \, of\; \, treated\; \, cells/Mean\; \, OD\; \, of \, \;untreated\; \, cells\; \, \left( {control} \right) \, \times \, 100.$$

### Statistical analysis

Results were expressed as mean ± SD of three independent experiments. Difference between groups was analyzed by Two way ANOVA from GraphPad Prism-6.0; ns = not significant; *p < 0.5, **p < 0.01 and ***p < 0.001 were considered statistically significant at the respective levels.

## Abbreviations

DMSO: Dimethyl sulfoxide
FUM: Fumagillin
GT: Gliotoxin
IC_50_: Concentration at which 50% of cells are dead
IdMOC: Integrated discrete multiple organ co-culture
IA: Invasive aspergillosis
MTT: 3-4, 5-Dimethylthiazole-2-yl, 2,5-diphenyl tetrazolium bromide
ROS: Reactive oxygen species
